# Superior Oxidase-Mimetic Activity of Co-MOF Nanozyme for Smartphone-Based Visually Colorimetric Assay of Mancozeb

**DOI:** 10.3390/molecules30244758

**Published:** 2025-12-12

**Authors:** Shuyue Pang, Lina Chen, Yangyuxin Liu, Xiuting Lu, Hongfei Liu, Yuting Shu, Helong Bai, Jing Wang, Dongfang Shi

**Affiliations:** 1 College of Chemistry, Changchun Normal University, Changchun 130032, China; 15567234211@163.com (S.P.); lyyx24@outlook.com (Y.L.); 19524312748@163.com (X.L.); qx202308010@ccsfu.edu.cn (H.L.); qx202408003@stu.ccsfu.edu.cn (Y.S.); baihelong@ccsfu.edu.cn (H.B.); 2 Central Laboratory, Changchun Normal University, Changchun 130032, China; chenlina4321@163.com; 3 Institute of Science and Technology Innovation, Changchun Normal University, Changchun 130032, China; 4 College of Life Sciences, Changchun Normal University, Changchun 130032, China

**Keywords:** Co-based nanozymes, smartphone, colorimetric analysis, paper-based sensors, Mancozeb

## Abstract

Mancozeb (MCZ), a widely used fungicide in agricultural production, has been reported as an environmental endocrine disruptor, posing serious risks to ecosystems and human health. In this work, multivalent Co-MOF nanozymes (MVCM) with excellent oxidase-like activity were synthesized, which can promote the oxidation of 2,2′-azino-bis-(3-ethylbenzothiazoline-6-sulfonic acid) (ABTS) into a blue oxidative product (ABTS^•+^), with an obvious absorption peak at 415 nm. With the addition of MCZ, the ABTS^•+^ was reduced to colorless ABTS through the REDOX reactions between MCZ and ABTS^•+^. Based on the unique reducing behavior of MCZ, a nanozyme-based colorimetric detection platform was proposed for the detection of MCZ, with a linear range of 3–27 μM and a detection limit (LOD) of 0.15 μM. Furthermore, the sensor was integrated with smartphones and test strips, establishing a portable smartphone-based platform for the real-time, on-site, and visual quantitative detection of MCZ. The detection concentration range was 15–90 μM, with LOD as low as 15 μM. The assay exhibited high adaptability in practical applications. In summary, this work provided a simple, accurate, and low-cost approach for visual determination of MCZ without complicated instruments and procedures.

## 1. Introduction

Mancozeb (MCZ) is an ethylene-bis-dithiocarbamate (EBDC) pesticide, which is widely used as a fungicide in protecting fruit and vegetable crops from fungal disease. The unreasonable or excessive use of MCZ can increase its residues in food and the environment, posing serious threats to human health [1]. Moreover, long-time exposure to MCZ causes a variety of toxic diseases, including apoptosis and neuroinflammation in hypothalamic neurosecretory cells [2], oxidative stress in lymphoid organs [3], and apoptosis in male germ cells. Therefore, it is critical to create efficient, affordable, and manageable methods for detecting MCZ. At present, a variety of analytical methods, such as flow injection (FLA) [4], high-performance liquid chromatography (HPLC) [5], gas chromatography (GC) [6], triple quadrupole mass spectrometry (MS) [7], and LC tandem mass spectrometry (LC-MS/MS) [6], which exhibit high sensitivity and good reproducibility, have been employed in the monitoring of MCZ in water, soil, and food samples. However, these chromatography-based methods are time-consuming, expensive, require trained operators, and are not suitable for on-site detection. Colorimetric-fluorescence and electrochemical methods have also been explored for the detection of MCZ [8,9]. These methods have attracted considerable attention owing to the advantages of simplicity, low cost, and visualization. However, their application in on-site monitoring of MCZ remains challenging due to the current requirement for bulky instrumentation and computer workstations that hinder portable deployment.

The paper-based sensor is an integrated analytical platform that combines sample pretreatment and detection processes, leveraging its inherent microfluidic channels and porous cellulose fiber structure to enable on-chip filtration and biochemical sensing of complex samples, thereby eliminating the need for external detection equipment. It is widely used in food safety, medical detection, environmental monitoring, and other fields due to its low cost, good biocompatibility, and environmental friendliness. But the results of this method are primarily qualitative or semi-quantitative due to insufficient sensitivity. With the emergence of integrated technologies based on intelligent devices and point-of-care testing (POCT) platforms, smartphone-assisted paper-based colorimetric sensors have garnered significant attention in the field of on-site pesticide detection as a novel portable analytical platform. This innovative technology, through the integration of three-channel red, green, and blue (RGB) color analysis, demonstrates remarkable operational convenience, rapid response characteristics, and in situ detection capabilities. It thereby provides an innovative solution for real-time quantitative detection of target analytes [10].

As is well known, enzymes are a promising candidate for creating a colorimetric analytical system because of their substrate specificity and outstanding catalytic performance. Natural enzymes are susceptible to degeneration by external influences, which restricts their applications. Nanozymes, which are nanomaterials that mimic natural enzyme activity, have gained considerable attention for their straightforward synthesis, stable performance, and ease of reconfiguration [11]. Doping and loading nanozymes onto the surfaces of metal–organic frameworks (MOFs) represent promising strategies for enhancing the activities of nanozymes [12], attributed to the outstanding surface areas and porous structures of MOFs that facilitate efficient mass transfer of reactants and intermediates during the catalytic processes [13]. Leveraging the large specific surface area of MOFs to accommodate a significant amount of oxide nanozymes, this approach increases the local concentration of the catalyst and utilizes their porous channels. This method is expected to provide a foundation for enhancing multi-mimic activity, thereby creating favorable conditions for catalysis and detection. Liu et al. [14] synthesized copper-doped cerium dioxide loaded on PCN-224 with enhanced multi-mimics through the in situ growth method (PCN-224@Cu-CeO_2_). Utilizing the OXD-like, AAO-like, and GPx-like activities of PCN-224@Cu-CeO_2_, TAC, AA, and GSH can be evaluated. Chen et al. [15] synthesized a nanomaterial (PCN-224@Au@MnO_2_@HA) with excellent peroxidase activity and constructed a tumor cell-targeting, GSH-activatable, and oxygen self-supplying nanoplatform to effectively inhibit tumor growth.

Recently, Co-based nanozymes have attracted extensive attention due to their superior physicochemical properties. The catalytic activity of Co_3_O_4_ nanozymes, which have emerged as prominent candidates in biomimetic catalysis, is principally governed by multiple structural parameters, including morphological characteristics, particle dimensions, dispersion homogeneity, and colloidal stability. Notably, structural degradation of Co_3_O_4_ nanozymes during catalytic cycles frequently leads to diminished catalytic efficiency. Comparative analyses of distinct morphological variants (e.g., nanoplates, nanorods, and nanocubes) reveal significant discrepancies in electron transfer kinetics and consequent catalytic performance, as documented in previous studies [16]. 3D ZIF-67 is a cobalt-based metal–organic framework (MOF) formed by the coordination of Co^2+^ with 2-methylimidazole ligands, demonstrating superior enzymatic activity compared to conventional cobalt-based nanozymes. This enhancement originates from its expanded specific surface area and improved accessibility to catalytically active sites [17,18]. Post-modified synthesis is a technique used to functionalize or modify materials through further chemical or physical treatments after the initial synthesis [19]. The goal is to optimize the material’s performance or impart new functionalities without altering the fundamental structure of the original material [20]. The term ‘MVCM’ was initially used to describe a cerium (Ce)-based mixed-valence nanozyme [21]. With the development of this type of material system, subsequent studies have also classified cobalt (Co)-based MOF materials with similar mixed valence state characteristics as MVCM [22]. To maintain consistency and coherence in the terminology system with existing research, this paper also uses ‘MVCM’ to refer to the synthesized cobalt-based mixed-valence MOF material. Recent advancements demonstrate that MVCM exhibits remarkable oxidase-mimetic activity, enabling efficient oxidation of the chromogenic substrate ABTS from its colorless native state to the blue radical cation (ABTS^•+^). The ABTS-MVCM chromogenic system’s distinctive colorimetric response facilitates the development of stimulus-responsive test strips. When integrated with smartphone-based analytical platforms, this technology shows significant promise for field-deployable detection systems, particularly in resource-limited environments.

**Figure 1:**
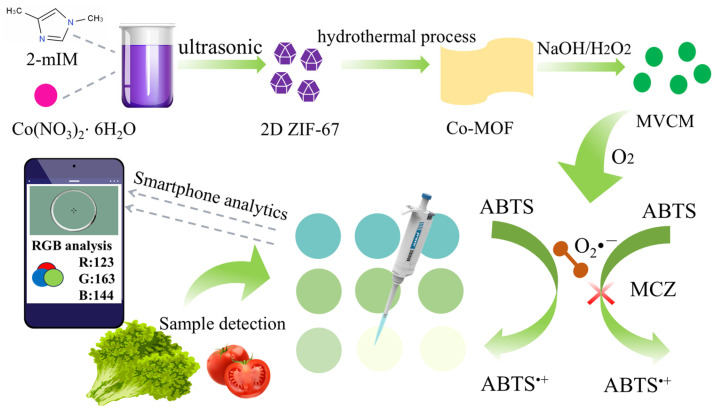
Schematic diagram of MVCM-based MCZ colorimetric sensor construction.

In this study, an “all-in-one” portable paper-based sensing platform integrated with a smartphone was proposed to realize rapid on-site detection of MCZ (Figure 1). The multivalent Co^2+^/Co^3+^ MOF nanozyme (MVCM) was successfully prepared via the hydrothermal method and immobilized on paper substrates, which can promote the oxidation of ABTS into the blue oxidative product (ABTS^•+^). With the increase in MCZ concentration, the blue color intensity of the solution decreased in a concentration-dependent manner. Based on the reduction mechanism of MCZ in the color reaction [8], we developed a simple and rapid intelligent visual detection platform for the detection of MCZ in food samples by integrating smartphone photography with a dedicated application (Color Picker) for colorimetric data acquisition and analysis. The integrated intelligent detection platform demonstrates considerable potential for practical applications in the rapid detection of MCZ.

## 2. Results and Analysis

### 2.1. Characterization of MVCM

**Figure 2:**
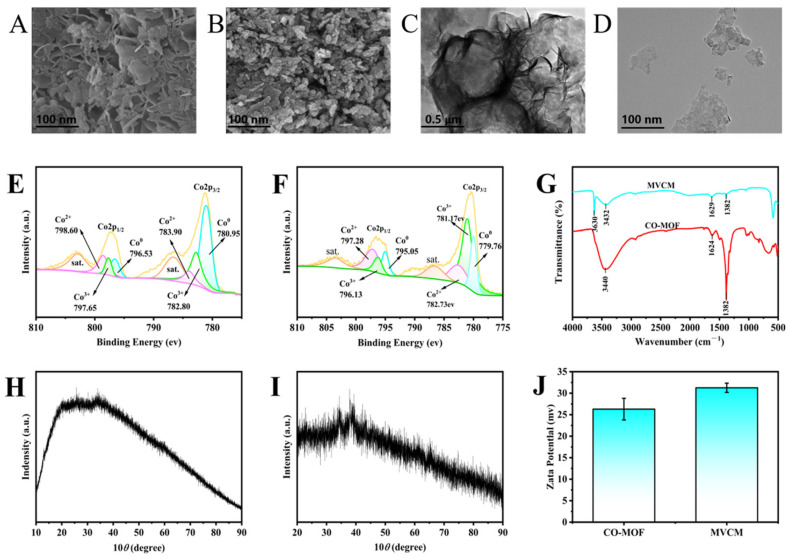
(**A**,**B**) SEM images of Co-MOF nanosheets and MVCM; (**C**,**D**) TEM images of Co-MOF nanosheets and MVCM; (**E**,**F**) Co2p XPS spectra of 2D Co-MOF nanosheets and MVCM; (**G**) FT-IR spectra of 2D Co-MOF nanosheets and MVCM; (**H**,**I**) XRD patterns of 2D Co-MOF nanosheets and MVCM; (**J**) Zeta potentials of 2D Co-MOF and MVCM.

Firstly, the morphology of Co-MOF and MVCM was observed by SEM and TEM. As shown in Figure 2A, the SEM image of Co-MOF showed a regular lamellar morphology with clearly visible edges. In contrast, the SEM image of MVCM (Figure 2B) shows a rough and irregular surface topography with a large number of holes and uneven three-dimensional structures. However, the TEM image in Figure 2C further confirmed the lamellar structural features of Co-MOF, with no obvious defects or agglomerations on the surface of the material and uniform layer spacing. The TEM image in Figure 2D shows some aggregation of MVCM nanoparticles. The morphological changes in the nanomaterials clearly indicate the successful formation of MVCM.

X-ray Photoelectron Spectroscopy (XPS) analysis further elucidated the elemental composition and chemical structure of MVCM (Figure 2F). The composition primarily included cobalt (Co, 50.44%), carbon (C, 10.38%), and oxygen (O, 30.35%). The high-resolution Co 2p spectrum showed two peaks at 781.07 and 797.28 eV, attributed to Co 2p_3/2_ and Co 2p_1/2_ [23], and two satellite peaks (denoted as “sat.”) located at 786.81 and 802.93 eV. The Co 2p_3/2_ peak can be further divided into three peaks at 780.95, 782.80, and 783.90 eV, which are assigned to Co^0^, Co^3+^and Co^2+^ [24], respectively. According to their characteristic band areas, the ratio of Co^3+^/Co^2+^ increased after 2D CO-MOF nanosheets were modified into MVCM, which proved the successful oxidation of 2D Co-MOF, and the content of trivalent cobalt increased. The chemical bonding states of the 2D MOF and MVCM were further elucidated via FTIR spectroscopy. As presented by the red curve in Figure 2G, the broad and strong bands centered at 3440 cm^−1^ and 1382 cm^−1^ correspond to the stretching vibrations O-H and C=O, respectively [25,26]. Compared with the red curve, the band at 3630 cm^−1^ in the blue curve is assigned to the characteristic peak of Co-OH stretching vibration [27]. The appearance of Co-OH peaks in the blue curve and the weakening of the O-H and C=O peaks in the red curve indicated that MVCM was synthesized successfully.

The X-ray diffraction (XRD) pattern of MVCM is shown in Figure 2I, demonstrating a spinel-type structure. The diffraction peaks at 34.73°, 38.65°, and 65.27° can be assigned to (311), (222), and (440) reflections. The crystal structures’ diffraction peaks confirmed the presence of a pure Co_3_O_4_ phase. The XRD pattern transformed from a wide peak (Figure 2H) with lower crystallinity to a characteristic peak with spinel Co_3_O_4_, indicating the successful oxidation of MVCM. This is consistent with the SEM and TEM results. Analysis of the zeta potentials of the investigated materials provided additional evidence for the successful formation of the target nanomaterial (Figure 2J). The potential of 2D Co-MOF is +26.3 mV. Upon oxidation, the potential of MVCM increased to +31.3 mV, more positive than that of 2D Co-MOF, further confirming the increased Co^3+^ content in MVCM.

### 2.2. The Oxidase-like Activity of MVCM

**Figure 3:**
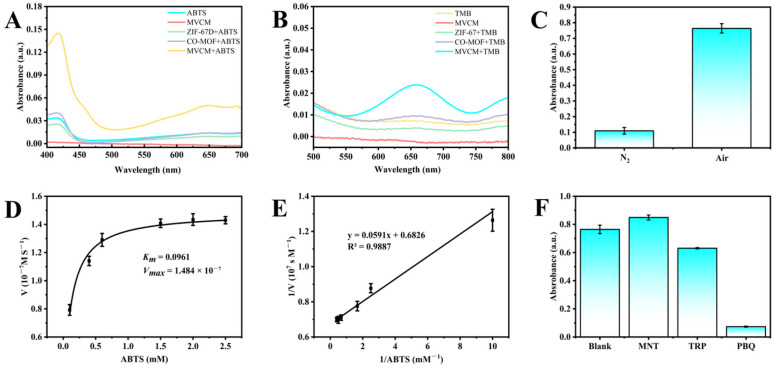
(**A**) UV–vis absorption spectra of different nanozymes reacting with ABTS; (**B**) UV–vis absorption spectra of different nanozymes reacting with TMB; (**C**) The oxidase-like activity of MVCM in the presence or absence of O2; (**D**) Michaelis–Menten curve and (**E**) corresponding Lineweaver–Burk plot of steady-state kinetics analysis for MVCM; (**F**) The effects of different radical scavengers on the absorbance of MVCM + ABTS reaction system.

Two chromogenic substrates, ABTS and TMB, were used to evaluate the oxidase-like activity of the prepared nanomaterials. ABTS is a negatively charged substrate that can be oxidized to ABTS^•+^, with a characteristic absorption peak at 415 nm and a change in color from colorless to blue-green. Based on the absorbance and color change, the oxidase-like activity of different nanomaterials can be evaluated [28]. As shown in Figure 3A, molecular oxygen (O_2_) was unable to directly oxidize ABTS, and the nanomaterial MVCM did not exhibit significant absorption interference at 415 nm. The addition of 3D ZIF-67 and 2D Co-MOF only triggered a weak ABTS oxidation reaction, which was manifested by negligible absorbance change and insignificant solution coloration. When MVCM was added, the catalytic system showed significant absorbance enhancement at 415 nm, accompanied by an obvious solution color change from colorless to characteristic green, which fully demonstrated the excellent oxidase-like catalytic activity of MVCM. The morphological transition from 2D to 3D significantly enhanced the oxidase-like activity of the nanozymes, indicating that this strategy can effectively enhance the catalytic performance of the nanomaterials.

To further verify the catalytic performance of the nanomaterials, TMB was also used as a typical chromogenic substrate for evaluation (Figure 3B). In the presence of the nanozymes, the colorless TMB could be oxidized by oxygen to blue oxTMB, with a significant absorption at 652 nm. Similarly to the results of the ABTS experiments, no increase in absorbance was detected in the presence of 3D ZIF-67. The addition of 2D Co-MOF and MVCM could catalyze the oxidation of TMB in small amounts. This may be due to the positively charged surface of MVCM and the electrostatic repulsion with the positively charged TMB, leading to a decrease in substrate adsorption efficiency. It is noteworthy that the absorption intensity of oxTMB by the nanomaterials was much lower than that of ABTS^•+^, which may be attributed to the fact that positively charged nanomaterials are more likely to bind to negatively charged ABTS. Based on the experimental findings and taking into account the superior detection sensitivity exhibited by ABTS, the ABTS chromogenic substrate was selected in conjunction with MVCM as the optimal detection system for subsequent experimental phases of this investigation.

### 2.3. Oxidase Simulation Mechanism of MVCM

Molecular oxygen (O_2_) is considered to be a key participant in the catalytic process of oxidative enzymes, and cobalt-containing MVCM itself possesses the ability to directly oxidize the substrate ABTS. To elucidate the specific action mechanism of MVCM in the oxidation process, we performed comparative experiments by incubating MVCM with ABTS in a nitrogen-rich environment. The experimental results showed that the characteristic absorption peak at 415 nm was significantly reduced under nitrogen-enriched conditions, and the solution was colorless (Figure 3C). This phenomenon suggests that O_2_ plays an indispensable role in the catalytic process of the mimetic oxidase, and MVCM acts as an oxidase-like enzyme mainly by catalyzing the oxidation of ABTS by O_2_ rather than functioning as a direct oxidant.

Literature studies have shown that dissolved oxygen can be converted into a variety of reactive oxygen species (ROS) and participate in the oxidation-catalyzed reaction process [29]. Among them, hydroxyl radical (•OH), singlet oxygen (^1^O_2_), and superoxide anion (O_2_^•−^) are common ROS types. In order to identify the main reactive oxygen species in the MVCM catalytic system, mannitol, tryptophan, and p-benzoquinone were used as specific trapping agents of •OH, ^1^O_2,_ and O_2_^•−^ in this study [28]. As shown in Figure 3F, after the addition of mannitol or tryptophan, the catalytic activity of ABTS oxidation catalyzed by MVCM was not significantly affected, indicating that the amount of •OH and ^1^O_2_ produced in the system was very small. However, the absorbance of the MVCM/ABTS system was significantly reduced when p-benzoquinone was added, confirming that O_2_^•−^ was the dominant active species in the catalytic reaction. In conclusion, MVCM nanomaterial mainly generates O_2_^•−^ by activating dissolved oxygen and then mediates the oxidation reaction of ABTS.

To further prove that the oxidase-like activity of MVCM originated from MVCM itself rather than from metal ions that might have been leached from the reaction system, we conducted XPS analysis and leaching experiments. The XPS spectrum indicates that a distinct cobalt element characteristic peak can be detected in the MVCM before the reaction, but no cobalt element signal is detected in the reaction solution (Figure S1). In addition, the MVCM was placed in the HAc-NaAC buffer solution (pH 3.0) and shaken vigorously for 2 h. After centrifugation, the supernatant of MVCM was used to catalyze ABTS to obtain the corresponding ultraviolet–visible absorption spectrum. The result demonstrates that ABTS is not oxidized by the supernatant, showing the prominent intrinsic oxidase mimetic activity of MVCM.

### 2.4. Steady-State Dynamics Analysis

In order to systematically evaluate the oxidase-like activity of MVCM, a steady-state kinetic study was carried out. Under optimized experimental conditions (pH 3.0, 2.5 mg/mL MVCM), different concentrations of ABTS were added, and the absorbance at 415 nm was recorded to obtain a typical Michaelis–Menten equation curve (Figure 3D). The *V_max_* and *K_m_* of MVCM were obtained by double inverse plots (Figure 3E). *K_m_* and *V_max_* were 0.0961 ± 0.0097 and (1.484 ± 0.02375) × 10^−7^ M s^−1^, respectively. This is not much different from the previously reported *K_m_* 0.057 mM, *V_max_* 4.728 × 10^−8^ M s^−1^ [22], confirming that it has a similar substrate affinity. The observed differences were within a reasonable range [21,30], mainly attributed to the batch-to-batch variations in the configuration of the chemically active sites on the surface of the nanozyme and the subtle influence of the measurement microenvironment. This reflects the inherent sensitivity of the kinetic parameters of nanozymes to the synthesis and determination conditions. In general, the *K_m_* values of different nanozymes for the substrates represent the affinity strength of the artificial nanozymes for the substrates. The lower the *K_m_* value, the stronger the affinity of the artificial nanozymes for the substrates. The comparison of the nanomaterials prepared in this study with other reported materials is listed in Table 1. Compared with other reported nanomaterials, MVCM has a relatively small *K_m_* and a relatively high *V_max_*, which suggests that MVCM is a promising oxidase-like nanozyme.

### 2.5. Optimization of Reaction Conditions

**Figure 4:**
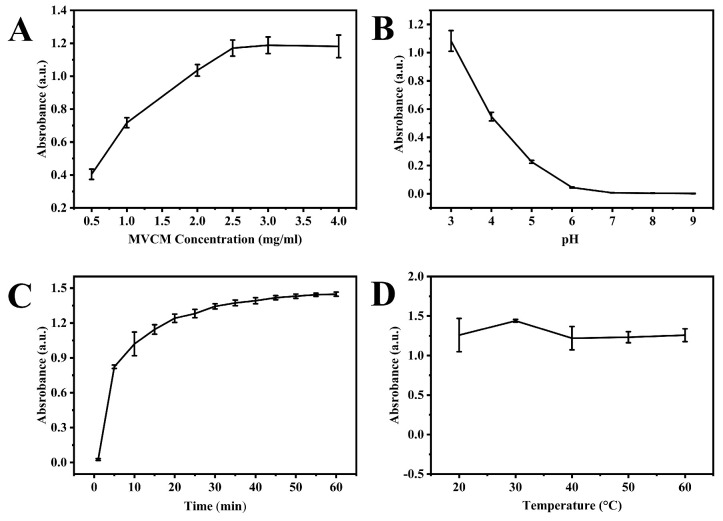
The effect of experimental condition on the oxidase-like activity of MCVM: (**A**) the concentration of MVCM (pH: 3.0, reaction time: 15 min, temperature: room temperature); (**B**) pH (the concentration of MVCM: 2.5 mg/mL, reaction time: 15 min, temperature: room temperature); (**C**) reaction time (the concentration of MVCM: 2.5 mg/mL, pH: 3.0, temperature: room temperature); (**D**) reaction temperature (the concentration of MVCM: 2.5 mg/mL, pH: 3.0, reaction time: 15 min).

The experimental parameters, such as MVCM concentration, pH, incubation time, and thermal conditions, were examined to improve the performance of the nanosensor. The results revealed that MVCM concentration significantly influenced the catalytic performance of MVCM. The maximum catalytic activity was achieved at a concentration of 2.5 mg/mL of MVCM (Figure 4A). Consistent with the characteristic pH-dependent behavior of most nanozymes, MVCM displayed pronounced pH sensitivity in oxidase-mimetic activity (Figure 4B). The catalytic efficiency peaked at pH 3.0 in HAc-NaAc buffer, showing a significant reduction in activity when the pH increased to neutral conditions. This strong acid dependence necessitated the selection of a pH 3.0 buffer as the standard reaction medium for subsequent experiments. Also, the effect of reaction duration on the oxidase-like activity of MVCM is shown in Figure 4C. The results suggested that the reaction between MVCM and the ABTS system increased sharply in the first 10 min and stabilized afterward at 40 min, indicating that the reaction reached near-completion within this time frame. The thermal effects were also optimized (Figure 4D). The oxidase-mimicking activity of MVCM remained virtually unchanged across a broad thermal range (20–60 °C). This temperature-independent behavior confirms the material’s exceptional thermal stability and robust structural integrity under varying thermal conditions. Considering both operational practicality and energy efficiency, all experimental procedures were subsequently standardized at ambient temperature (25 °C).

### 2.6. Colorimetric Detection of MCZ

**Figure 5:**
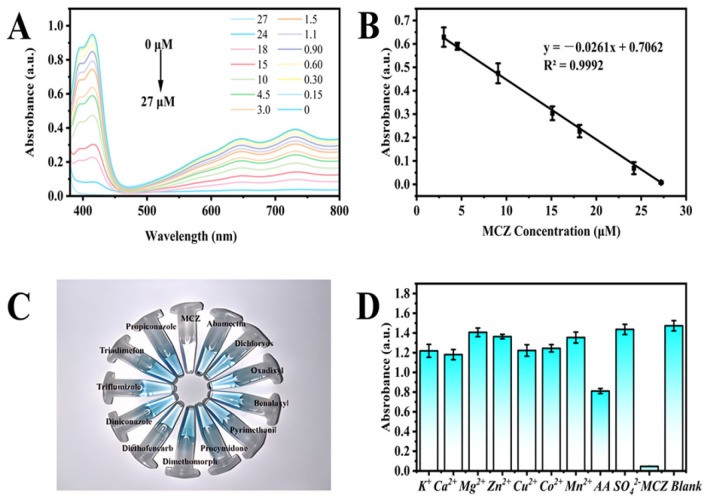
(**A**) UV–vis absorption spectrum of MVCM + ABTS system in the presence of MCZ; (**B**) The relationship between UV–vis absorption and the concentration of MCZ; (**C**) Corresponding color of the solutions. The concentration of each pesticide is 1 mg/mL; (**D**) UV–vis absorbance of the MVCM + ABTS system at 415 nm in the presence of MCZ or other different substances with a concentration of 20 μM, respectively.

A convenient and rapid colorimetric analysis platform was established using the oxidase-like activity of MVCM. Varying concentrations of MCZ (0.15–27 μM) were introduced into the MVCM-based colorimetric sensor, and the corresponding changes in its UV–vis absorption spectra were recorded. As shown in Figure 5A, the absorption band at 415 nm significantly decreased with the increase in MCZ concentration. Simultaneously, the solution changed from blue to colorless. Under the optimal experimental condition, a linear relationship between UV–vis absorption and MCZ concentrations was established, as shown in Figure 5B. The linear regression equation of MCZ in colorimetric detection is y = −0.0216x + 0.7062 (R^2^ = 0.9992), and the detection limit (LOD) is 0.15 μM, which was superior to the detection performance of the conventional nanozyme cascade sensor (Table 2). Firstly, the method not only eliminates the dependence on unstable and costly acetylcholinesterase (AChE) but also shows excellent selectivity for some pesticides and coexisting substances (Figure 5C,D). Secondly, the colorimetric detection constructed using the oxidase-like activity of MVCM avoids the use of H_2_O_2_, thereby enhancing the reliability and stability of the detection signal. Further comparison with instrumental methods reveals that the method exhibits a relatively wide detection range, a lower detection limit, is simpler to operate, and is less expensive (Table 3). Overall, the MVCM-based colorimetric sensor is a potential candidate for MCZ detection.

### 2.7. Mancozeb Detection by Smartphone-Test Paper Platform

**Figure 6:**
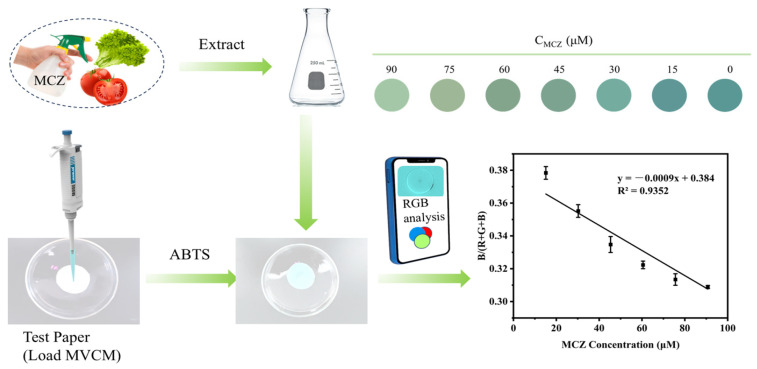
Schematic illustration of mancozeb detection using the smartphone-test paper platform and linear relationship between the B/(R+G+B) value and mancozeb concentrations within the range of 15–90 mM based on color change.

A test smartphone-integrated paper-based analytical platform incorporating MVCM was developed for real-time monitoring of MCZ. A series of MCZ solutions with concentration gradients was added to the paper-based sensor. Subsequently, chromatic data derived from the colorimetric images were quantitatively transformed into digital RGB parameters through chromatic digitization, thereby establishing a foundation for correlative concentration quantification. As illustrated in Figure 6, the color intensity of the test paper gradually became lighter with increasing MCZ concentration, and a good linear relationship between B/(R+G+B) values and MCZ concentration in the range of 15–90 μM has been obtained. The linear relationship is expressed as B/(R+G+B) = −0.0009x + 0.384 (*R*^2^ = 0.9352). The integrated detection platform exhibits portability and visualization capabilities, enabling real-time on-site detection of MCZ.

### 2.8. Real Sample Analysis

To validate the practicality and reliability of the developed colorimetric sensor, lettuce and tomato were employed as real samples for MCZ detection (Table 4), and the UV–vis detection method was used for comparison. In accordance with the method’s detection range and potential analyte loss during sample pretreatment, four spiked levels (15, 30, 60, and 90 μM) were established for vegetable analysis. The recoveries ranged from 82.17% to 109.63% with relative standard deviations (RSD) not exceeding 5.82%. These precision parameters comply with the quantitative detection criteria specified in the Chinese agricultural standard NY/T 788-2018 [46]. The limits of quantification (LOQ) of lettuce and tomato were 1 mg/kg, meeting the requirements of China’s current GB 2763-2021 [47] for the quantitative detection of MCZ residues. Comparatively, the obtained results indicated that our method had a lower detection limit and comparable accuracy to that of the UV–vis method. This result confirms that the smartphone colorimetric sensor based on MVCM exhibits satisfactory accuracy and feasibility in the on-site detection of real samples and provides a reliable analytical method for the rapid detection of pesticide residues in food.

## 3. Materials and Methods

### 3.1. Materials and Reagents

Unless otherwise stated, all compounds and reagents were purchased and used directly without purification. Cobalt nitrate hexahydrate (Co (NO_3_)_2_ 6H_2_O) and 2-methylimidazole (2-mIM) (Aladdin Biochemical Technology Co., Ltd., Shanghai, China). 2,2′-azino-bis-(3-ethylbenzothiazoline-6-sulfonic acid) (ABTS), 3,3′,5,5′-tetramethylbenzidine (TMB), and acetic acid buffer solution (HAc-NaAc, pH 3.0) (Yuanye Biotechnology Co. Ltd., Shanghai, China). Sodium hydroxide (NaOH), absolute ethanol (C_2_H_5_OH), hydrogen peroxide (H_2_O_2_, 30%), and methanol (CH_3_OH) (Fuyu Fine Chemical Co., Ltd., Tianjin, China). Mancozeb (99.6%) (Sigma. Co., Ltd., Shanghai, China). All reagents used in the work were AR grade, and pure water (18.2 MΩ cm) was used in all experiments.

### 3.2. Instruments and Equipment

The crystal, morphological structure, and elemental composition were analyzed using X-ray diffraction (XRD, ultimaIV, Rigaku, Akishima-shi, Japan), scanning electron microscopy (SEM, Zeiss sigma300, Oberkochen, Germany), X-ray photoelectron spectroscopy (XPS, ESCALAB-250Xi, Thermo Fisher Scientific, Waltham, MA, USA), and transmission electron microscopy (TEM, FEI Tecnai F20, Hillsboro, OR, USA). UV–vis absorption spectra were obtained by a UV–Vis spectrophotometer (UV-2200, Beijing Beifen-Ruili Analytical Instrument (Group) Co., Ltd., Beijing, China); infrared spectra (IR) were acquired on a Nicolet Simmit FT-IR spectrometer (Thermo Fisher, Waltham, MA, USA); Zeta potentials of different materials were obtained on Zetasizer (Zetasizer Nano ZS90, Malvern Instruments Ltd., Worcestershire, UK).

### 3.3. Preparation of MVCM

The preparation method referred to the previous reports [22,48,49]. Take 5 mL of 36.5 mg/mL cobalt nitrate hexahydrate and slowly add it to the 2-methylimidazole solution (41 mg/mL, 5 mL), ultrasonicate for 30 min, centrifuge, wash the precipitate three times with anhydrous methanol, and dry it in an oven at 40 °C to obtain ZIF-67 crystals. Disperse the ZIF-67 crystals in 5 mL of anhydrous methanol and add cobalt nitrate hexahydrate (5 mL, 36.5 mg/mL), mix well, transfer to a Teflon-lined stainless-steel autoclave, and store at 120 °C for 60 min. Then, centrifuge, wash the precipitate three times with anhydrous methanol, and dry to obtain yellow Co-MOF nanosheets. After dispersing 20 mg of Co-MOF nanosheets in 4 mL of ultrapure water, add 1 mL of the mixed solution freshly prepared from 950 μL of 2.5 M NaOH and 50 μL of 30% H_2_O_2_, shake for 2 min, then centrifuge to separate, and wash the precipitate with pure water until the supernatant is neutral. After drying, the final product is obtained and labeled as MVCM (multivalent Co-MOF).

### 3.4. Study on the Oxidase-like Activity of MVCM

To qualitatively verify and compare the intrinsic oxidase-like activity of this research material, a 10 min reaction incubation time was set for the experiment. This duration is a conventional choice for preliminary activity screening [48,50], sufficient to generate a significant spectral signal to distinguish the strength of activity. Specifically, 10 μL of MVCM was added to the mixed system of 975 μL of HAc-NaAC buffer solution (pH 3.0) and 15 μL of ABTS (4 mM), and after reacting for 10 min, the absorbance of ABTS^•+^ was immediately monitored at a wavelength of 415 nm using an ultraviolet–visible spectrophotometer to evaluate catalytic activity [51]. For the free radical scavenging experiments [52], 100 μL of a 20 mM free radical or electron scavenger, such as mannitol (MNT), tryptophan (TRP), and p-benzoquinone (PBQ), was added to the MVCM-ABTS reaction system, and the absorbance of ABTS^•+^ at 415 nm was recorded.

### 3.5. Steady-State Kinetics Study on MVCM

The oxidase-like activity of MVCM was determined at a fixed sample concentration (2.5 mg/mL) and different substrate concentrations (0–2.5 mM), and the total volume of 1 mL was controlled with HAc-NaAc buffer (pH 3.0). The change in absorbance at 415 nm (the characteristic absorption peak of ABTS^•+^ under acidic conditions) over time was recorded to monitor the color change in the reaction in time-scan mode. The Lambert–Beer law is utilized to determine the initial rate of reaction by Equation (1):

(1) $$v=\frac{k}{\varepsilon b}$$

(*k* = ΔA/Δt, *ε* = 36,000 M^−1^ cm^−1^, and *b* = 1 cm) By plotting the calculated initial rate versus substrate concentration, a Michaelis−Menten curve [28] is obtained. Equation (2):

(2) $$v=\frac{Vmax\times \left[S\right]}{Km+\left[S\right]}$$

In the equation, *v* represents the reaction rate, *Vmax* represents the maximum reaction rate at infinite substrate concentration, [S] represents the substrate concentration, and *K_m_* represents the enzyme’s substrate affinity constant. The Michaelis–Menten equation reveals a linear relationship between reaction rate and substrate concentration at low substrate concentrations, while the reaction rate approaches the maximum value *V_max_* as substrate concentration increases to *K_m_*. The Lineweaver–Burk equation is a linearization of the Michaelis–Menten equation, expressed as in Equation (3):

(3) $$\frac{1}{v}=\frac{Km}{Vmax}\times (\frac{1}{\left[S\right]}+\frac{1}{Km})$$

Plotting the reciprocal of the Michaelis–Menten equation yields a straight line with an intercept of 1/*V_max_* and a slope of *K_m_/V_max_*. The values of *K_m_* and *V_max_* can be calculated to evaluate the substrate affinity and catalytic activity of MVCM.

### 3.6. Colorimetric Assay for MCZ

Different concentrations of MCZ were added to HAc-NaAc buffer (pH 3.0) containing ABTS (4 mM, 15 μL) and MVCM (2.5 mg/mL, 15 μL). The system was incubated at room temperature for 40 min, and the absorbance (415 nm) of the MVCM system was measured. A standard curve was established based on the concentration of MCZ and the absorbance at 415 nm.

### 3.7. Selectivity and Anti-Interference Ability Analysis

ABTS (15 μL, 4 mM) and MVCM (2.5 mg/mL, 15 μL) were added to HAc-NaAc buffer solution (950 μL, pH 3.0) by addition of different pesticides (propiconazole, triadimefon, triflumizole, diniconazole, diethofencarb, dimethomorph, procymidone, pyrimethanil, benalaxyl, oxadixyl, dichlorvos, abamectin) and different interferents (K^+^, Ca^2+^, Mg^2+^, Zn^2+^, Cu^2+^, Co^2+^, Mn^2+^, amino acid, SO_4_^2−^) to test the selectivity and interference resistance of the MVCM sensor.

### 3.8. Visual Detection of MCZ Based on Smart Phone

The MVCM solution (15 uL, 2.5 mg/mL) was loaded on the filter paper with a diameter of 1 cm for preparing the detection test paper and then air-dried at room temperature for 30 min to create the detection platform. Subsequently, the paper was treated with 15 μL of HAc-NaAc buffer (pH 3.0) containing 4 mM ABTS. After 10 min of reaction, 20 μL of different concentrations of MCZ solution were added to the platform, and the color change on the test paper was observed after 5 min of reaction. The results of different concentrations of the MCZ reaction system were analyzed by color values with the help of the Color Picker application for smartphones.

### 3.9. Analysis of Actual Samples

Lettuce and tomato were selected as real samples to validate the feasibility of the assay. They were purchased from local supermarkets and preprocessed according to the methods described in the literature [53] with slight modifications. Specifically, samples were sprayed with different concentrations of MCZ, cut into small pieces of about 1 cm, and homogenized into a pulp using a homogenizer. Then, the pulp (2 g) was further diluted 10 times using a water–methanol mixture (6:4, *v/v*) to prepare the sample solution, which was incubated at room temperature for 1 h. Subsequently, the mixture was centrifuged at 12,000 rpm for 10 min and filtered through a 0.45 μm membrane to obtain the test solution. Finally, the concentration of MCZ was determined with the smartphone-assisted test paper probe following the procedure described in Section 3.8.

## 4. Conclusions

MVCM nanocomposites exhibiting exceptional oxidase-mimetic activity were successfully synthesized through a rational synthesis strategy in this study. It is noteworthy that systematic studies have shown that MCZ causes significant changes in the UV–visible spectrum by reducing ABTS^•+^ to ABTS, accompanied by a distinct color change from blue to colorless. Based on this redox mechanism, we established an innovative enzyme-free colorimetric detection platform that enables selective detection of MCZ with a detection limit of 0.15 μM. Furthermore, we pioneered the integration of this nanozyme-based colorimetric system with smartphone-assisted digital imaging, constructing an intelligent, field-deployable detection platform. The Color Picker application algorithmically processes RGB values extracted from camera-captured reaction images, enabling quantitative MCZ determination within 5 min through linear correlation (R^2^ = 0.9352) between chromaticity parameters and analyte concentration. Validation experiments demonstrated satisfactory recovery rates (82.17–109.63%) in spiked real samples, confirming method reliability for practical applications, indicating significant application potential in food safety monitoring and environmental pollution screening. Notably, the proposed mobile sensing integration strategy establishes an important technical paradigm for developing next-generation portable biosensors, effectively addressing the longstanding dependence of conventional methods on specialized instrumentation and complex operational procedures.

## Figures and Tables

**Table 1:** Steady-state kinetic parameters of different nanozymes.

Nanozymes	*K_m_*/(mM)	*V_max_*/(10^−8^ M S^−1^)	Reference
Heme-AuNPS	0.168	1.68	[31]
Mn_2_O_3_	0.130	15	[32]
Co_3_O_4_ NPS	0.051	3.3	[33]
Fe_3_O_4_	0.370	2.60	[34]
Fe-N-C SAzyme	0.253	4.11	[35]

**Table 2:** Detection limits of different nanozymes.

Nanomaterials	Methods	Detection Limit (μM)	Reference
CoPOM/AgNP	electrochemical	100	[36]
Cd-MOF	luminous	2.09	[37]
Co-CQDs/Fe^3+^	luminous	18	[38]
Co_3_O_4_	electrochemical	1	[39]
S-CQDs/CuNCs	luminous	7.04	[40]

**Table 3:** Detection limits of MCZ by different analytical methods.

Methods	Linear Range	Detection Limit	Reference
HPLC-DAD	0.5–9.3 mg/kg	0.04 mg/kg	[41]
voltammetry	10–90 μM	7 μM	[42]
electrochemical method	25–150 μM	10 μM	[43]
colorimetric method	50–300 μM	21.1 μM	[44]
flow injection chemiluminescence	0.0092–0.9218 μM	0.003 μM	[45]

**Table 4:** Determination of MCZ in real samples (*n* = 3).

Sample	Added (μM)	UV–vis Spectroscopy			Smartphone-Based Colorimetric Method		
		Found (μM)	RSD (%)	Recovery (%)	Found (μM)	RSD (%)	Recovery (%)
**Lettuce**	15	14.71	0.23	98.11	15.18	1.18	101.21
	30	29.79	0.85	99.30	29.68	2.50	98.95
	60	60.84	1.16	101.41	49.30	5.82	82.17
	90	85.27	1.06	94.75	88.46	1.03	98.29
**Tomato**	15	14.92	1.15	99.50	15.23	3.21	101.54
	30	30.72	0.51	102.41	27.77	2.57	92.56
	60	64.26	3.76	107.11	65.83	3.70	109.71
	90	100.07	0.92	111.18	98.66	5.46	109.63

## Data Availability

The original contributions presented in this study are included in the article/Supplementary Materials. Further inquiries can be directed to the corresponding author(s).

## Supplementary Materials

The following supporting information can be downloaded at: https://www.mdpi.com/article/10.3390/molecules30244758/s1, Figure S1: XPS spectra of MVCM and MVCM-ABTS reaction systems; Figure S2: UV-vis absorption spectra of different reaction systems. The reaction systems are composed with HAc- NaAc buffer solution (pH 3.0), 2.5 mg·mL^−1^ MVCM or the supernatant of MVCM and 4 mM ABTS.
