# Supplementary material for: Superior Oxidase-Mimetic Activity of Co-MOF Nanozyme for Smartphone-Based Visually Colorimetric Assay of Mancozeb
Source: Molecules. 2025 Dec 12;30(24):4758. doi: 10.3390/molecules30244758 (PMC12735412; doi:10.3390/molecules30244758)
Supplement: Supplementary file 1 [file molecules-30-04758-s001.zip › molecules-3961128-supplementary.pdf]

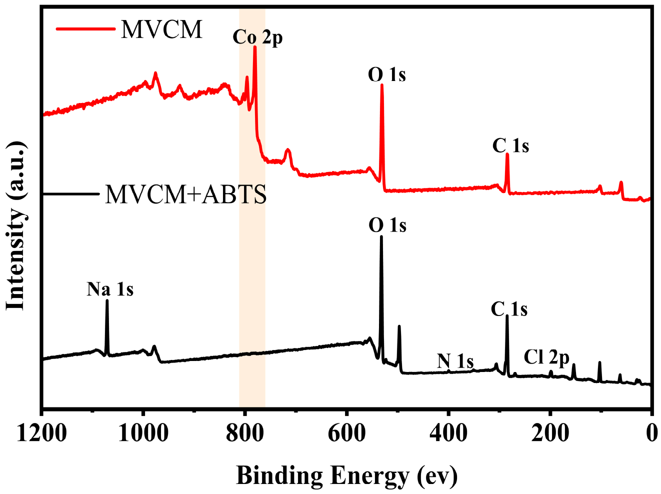

Figure S1. XPS spectra of MVCM and MVCM-ABTS reaction systems

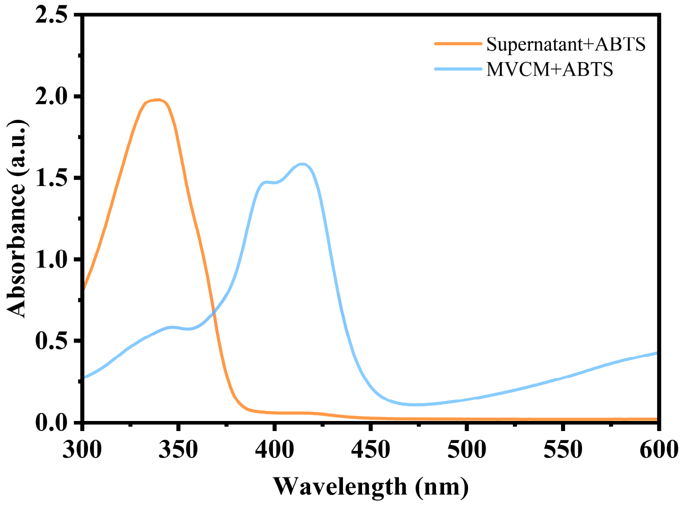

Figure S2. UV-vis absorption spectra of different reaction systems. The reaction systems are composed with HAc- NaAc buffer solution (pH 3.0), 2.5 mg·mL^{-1} MVCM or the supernatant of MVCM and 4 mM ABTS.
